# 63438 Differential chromatin accessibility at dorsal root ganglia enhancers is associated with nerve injury

**DOI:** 10.1017/cts.2021.414

**Published:** 2021-03-30

**Authors:** Kimberly E. Stephens, Weiqiang Zhou, Zhicheng Ji, Hongkai Ji, Yun Guan, Sean D. Taverna

**Affiliations:** 1 Department of Pediatrics, University of Arkansas for Medical Sciences and the Arkansas Children’s Research Institute; 2 Department of Biostatistics, School of Public Health, Johns Hopkins University; 3 Department of Biostatistics and Bioinformatics, Duke University; 4 Departments of Anesthesia and Critical Care Medicine and Neurological Surgery, School of Medicine, Johns Hopkins University; 5 Department of Pharmacology and Molecular Sciences, and the Center for Epigenetics, School of Medicine Johns Hopkins University

## Abstract

ABSTRACT IMPACT: Our improved understanding of the changes in chromatin accessibility that occur in persistent pain states may identify regulatory genomic elements that play essential roles in modulating gene expression in the DRG. OBJECTIVES/GOALS: Efforts to understand genetic variability involved in an individual’s susceptibility to persistent pain support a role for upstream regulation by epigenetic mechanisms. Our objective was to examine the transcriptomic and epigenetic basis of persistent pain following nerve injury. METHODS/STUDY POPULATION: We used a multiomic approach to identify novel molecular pathways associated with nerve injury-induced pain hypersensitivity. Adult Sprague Dawley rats were randomized to Chronic Constriction Injury (CCI) to the sciatic nerve or no treatment (naive). The ipsilateral L4-L6 dorsal root ganglia (DRG)s were removed on Day 14 and used for ChIP-seq for H3K4me1, ATAC-seq, and RNA-seq. We assessed for differential chromatin accessibility, transcription factor motifs, and enrichment for biological processes in chromatin accessible regions associated with cis-regulatory regions identified by ATAC-seq and H3K4me1 enrichment. Luciferase assays determined the functional significance of these sequences. RESULTS/ANTICIPATED RESULTS: We identified 58,446 genomic regions where H3K4me1 enrichment overlapped with chromatin accessibility. Differential analysis identified 2145 of these 58,446 regions that had changes in accessibility after CCI. The majority of these regions were located in introns or intergenic regions. Functional annotation of the differentially accessible regions identified disparate molecular functions enriched following nerve injury which suggests that altered chromatin structure plays a role in the development of mechanical hypersensitivity. Motif analysis identified specific transcription factor families whose binding sequences were enriched in regions of increased or decreased accessibility. Luciferase assays showed significant enhancement or repression of gene transcription. DISCUSSION/SIGNIFICANCE OF FINDINGS: Our data provides a comprehensive map of chromatin accessibility changes in the DRG after CCI and emphasizes the importance of chromatin structure in the development and maintenance of chronic pain.

